# Similarities and differences between SUNCT and SUNA: a cross-sectional, multicentre study of 76 patients in China

**DOI:** 10.1186/s10194-022-01509-6

**Published:** 2022-10-26

**Authors:** Shuhua Zhang, Ya Cao, Fanhong Yan, Sufen Chen, Wei Gui, Dongmei Hu, Huanxian Liu, Hongjin Li, Rongce Yu, Dan Wei, Xiaolin Wang, Rongfei Wang, Xiaoyan Chen, Mingjie Zhang, Ye Ran, Zhihua Jia, Xun Han, Mianwang He, Jing Liu, Shengyuan Yu, Zhao Dong

**Affiliations:** 1 School of Medicine, Nankai University, Tianjin, 300071 China; 2 Department of Neurology, the First Medical Center, Chinese PLA General Hospital, Beijing, 100853 China; 3 International Headache Centre, Department of Neurology, Chinese PLA General Hospital, Beijing, 100853 China; 4 Department of Neurology, Linyi Jinluo Hospital, Linyi, Shandong China; 5 Department of Neurology, Changsha Central Hospital Affiliated to University of South China, Changsha, Hunan China; 6 Department of Neurology, The First Affiliated Hospital of USTC, Hefei, Anhui China; 7 Department of Neurology, The Second Affiliated Hospital of Shandong First Medical University, Taian, Shandong China; 8 Department of Neurology, Dong E Hospital, Liaocheng, Shandong China; 9 Department of Neurology, People’s Hospital of Luanchuan, Luoyang, Henan China; 10 Department of Neurology, Wuhan No.1 Hospital of China Hubei Province, Wuhan, Hubei China

**Keywords:** SUNCT, SUNA, Demographics, Clinical characteristics

## Abstract

**Background:**

Short-lasting unilateral neuralgiform headache attacks with conjunctival injection and tearing (SUNCT) and short-lasting unilateral neuralgiform headache attacks with cranial autonomic symptoms (SUNA) have not been evaluated sufficiently due to limited data, particularly in China.

**Methods:**

Patients with SUNCT or SUNA treated in a tertiary headache centre or seven other headache clinics of China between April 2009 and July 2022 were studied; we compared their demographics and clinical phenotypes.

**Results:**

The 45 patients with SUNCT and 31 patients with SUNA had mean ages at onset of 37.22 ± 14.54 years and 42.45 ± 14.72 years, respectively. The mean ages at diagnosis of SUNCT and SUNA were 41.62 ± 12.70 years and 48.68 ± 13.80 years, respectively (*p* = 0.024). The correct diagnosis of SUNCT or SUNA was made after an average of 2.5 (0–20.5) years or 3.0 (0–20.7) years, respectively. Both diseases had a female predominance (SUNCT: 1.14:1; SUNA: 2.10:1). The two diseases differed in the most common attack site (temporal area in SUNCT, *p* = 0.017; parietal area in SUNA, *p* = 0.002). Qualitative descriptions of the attacks included stabbing pain (44.7%), electric-shock-like pain (36.8%), shooting pain (25.0%), and slashing pain (18.4%). Lacrimation was the most common autonomic symptom in both SUNCT and SUNA patients, while eyelid oedema, ptosis, and miosis were less frequent. Triggers such as cold air and face washing were shared by the two diseases, and they were consistently ipsilateral to the attack site.

**Conclusions:**

In contrast to Western countries, SUNCT and SUNA in China have a greater female predominance and an earlier onset. The shared core phenotype of SUNCT and SUNA, despite their partial differences, suggests that they are the same clinical entity.

## Introduction

Short-lasting unilateral neuralgiform headache attacks with conjunctival injection and tearing (SUNCT) and short-lasting unilateral neuralgiform headache attacks with cranial autonomic symptoms (SUNA) have been investigated from diverse perspectives, but both are regarded as unusual conditions among trigeminal autonomic cephalalgias (TACs) [1]. SUNCT was first described in 1978 by Sjaastad et al. [2], and the diagnostic criteria were formally established in 2004, in the second edition of the International Classification of Headache Diseases (ICHD-2) [3]. In the ICHD-2 appendix, SUNCT was categorised as a subset of SUNA as some patients do not have both conjunctival injection and tearing despite otherwise fulfilling the criteria for SUNCT. The two diseases were classified as one entity, short-lasting unilateral neuralgiform headache attacks (SUNHA), in both the ICHD third edition, version β (ICHD-3β) and ICHD third edition (ICHD-3), but with recognition of their distinct cranial autonomic symptoms [1, 4].

Earlier studies have explored the characteristics of, and relationship between, SUNCT and SUNA [5–18]. Some specialists have suggested that the classifications should be revised. Lambru et al. used new diagnostic criteria to merge the two diseases; it was suggested that the conditions differed simply in terms of the extent of cranial autonomic symptoms [11]. However, more cohorts from other regions require evaluation. Few such studies have been reported from China. As the first multicentre clinical cohort study of SUNCT and SUNA in China, this study characterised and analysed the demographics and clinical phenotypes of patients with SUNCT and SUNA. A comparison of our results with those of previous, representative clinical series contributes to a better understanding of the relationship between SUNCT and SUNA.

## Methods

This multicentre cross-sectional study was conducted at the International Headache Centre, Department of Neurology, of the Chinese PLA General Hospital and at seven other headache clinics between April 2009 and July 2022. The study was approved by the Ethics Committee of the Chinese PLA General Hospital and complied with the World Medical Association’s Declaration of Helsinki.

### Participants

Patients diagnosed with SUNCT or SUNA based on the ICHD-2, ICHD-3β, or ICHD-3 were recruited for study participation. Patients diagnosed with secondary headaches or those in whom SUNCT or SUNA could not be distinguished from other types of headache were excluded. Two headache specialists confirmed each ICHD-3 diagnosis. All patients provided informed consent before their inclusion in the study.

### Data collection

The multicentre clinical data were standardised and collected using a comprehensive semi-structured questionnaire. The questionnaire included detailed demographic information (e.g. sex, age, height, and weight), headache-related information (e.g. age at onset, duration of misdiagnosis, diagnosis made on previous clinical assessments, lifestyle, personal and family histories), clinical characteristics, including laterality, location (within and outside the trigeminal distribution region), quality (stabbing pain, electric-shock-like pain, shooting pain, and slashing pain), visual analogue scale (VAS) to measure pain severity (0–10, with 0 = no pain, and 10 = very severe pain), the duration and frequency of attacks, accompanying cranial autonomic symptoms and other symptoms (e.g. nausea, vomiting, photophobia, phonophobia), trigger factors, aggravating factors, the presence of refractory period and treatment effects.

### Statistical analysis

Statistical analyses were performed using SPSS (version 23.0; SPSS, Chicago, IL, USA) and the R Programming Language (version 3.6.2). Measurement data are expressed as means ± standard deviation or as medians with the interquartile range. Count data are expressed as numbers (percentage). The baseline characteristics of the SUNCT and SUNA cohorts were compared by χ2 test or Fisher’s exact test for categorical variables and by Student’s *t*-test or the Mann–Whitney *U-*test for continuous variables, depending on the distribution. A two-sided *p*-value < 0.05 was considered to indicate statistical significance.

## Results

### Demographics

**Fig. 1:**
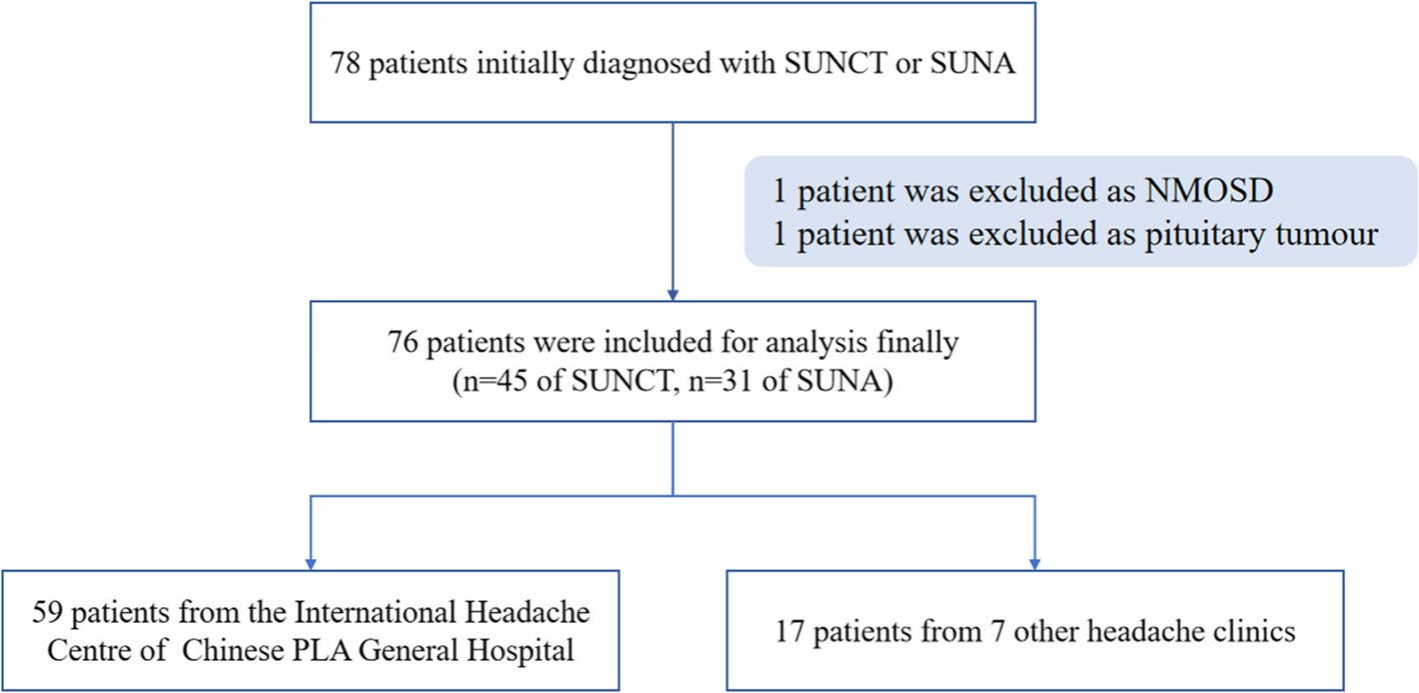
Flow chart of patient enrollment

**Fig. 2:**
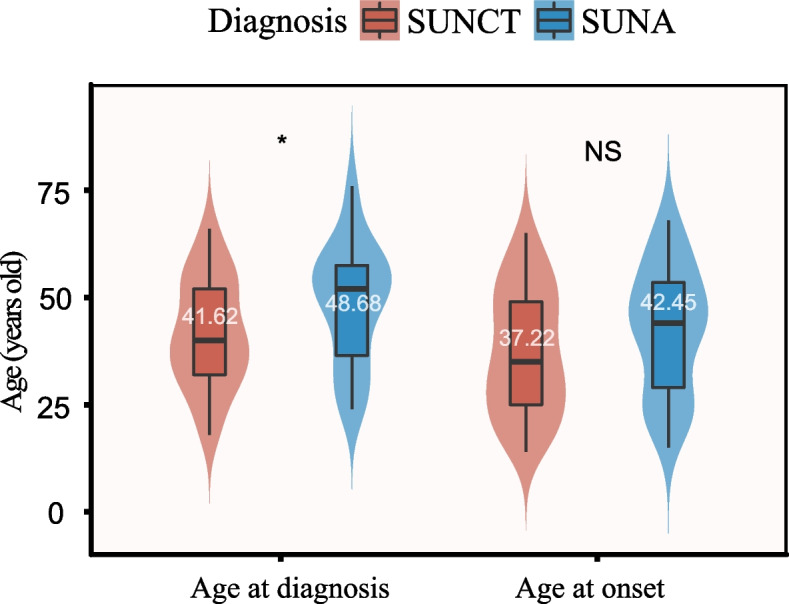
The left shows the distribution of age at diagnosis in patients with SUNCT and SUNA; the right shows the distribution of age at onset in patients with the two diseases

Among the 78 patients initially enrolled in the study, 2 were excluded as they were secondary to neuromyelitis optica spectrum disorders (NMOSD) and pituitary tumour, respectively, leaving 76 patients included in the analysis (Fig. 1). Of these, 45 (59.2%) patients, with a mean age of 37.22 ± 14.54 years (range: 14–65 years), were diagnosed with SUNCT, and 31 (40.8%) patients, with a mean age of 42.45 ± 14.72 years (range: 15–68 years), were diagnosed with SUNA (*p* = 0.024). The mean age at diagnosis was 41.62 ± 12.70 years (range: 18–66 years) in the SUNCT patients and 48.68 ± 13.80 years (range: 24–76 years) in the SUNA patients (*p* = 0.024) (Table 1, Fig. 2). In both the SUNCT and SUNA cohorts, there was a female predominance, with male: female ratios of 1:1.14 and 1:2.10, respectively (*p* = 0.209) (Table 1).

**Table 1:** Demographics and clinical characteristics of the study participants (*n* = 76)

Characteristics	SUNCT n (%)	SUNA n (%)	Total n (%)	*p* Value (< 0.05)
**Sex, n (%)**				0.209
** Male**	21 (46.7)	10 (32.3)	31 (40.8)	
** Female**	24 (52.2)	21 (67.7)	45 (59.2)	
**Age at diagnosis, Mean (SD), y**	41.62 (12.70)	48.68 (13.80)	44.50 (13.53)	0.024*
**Age at onset, Mean (SD), y**	37.22 (14.54)	42.45 (14.72)	39.36 (14.74)	0.129
**Median diagnostic delay time, IQR, y**	2.50 (0–20.5)	3.00 (0–20.7)	2.67 (0–20.7)	0.536
**Site, n (%)**				
** Periorbital**	13 (28.9)	4 (12.9)	17 (22.4)	0.100
** Forehead**	11 (24.5)	6 (19.4)	17 (22.4)	0.601
** Temporal**	24 (53.3)	8 (25.8)	32 (42.1)	0.017*
** Parietal**	13 (28.9)	20 (64.5)	33 (43.4)	0.002*
** Occipital**	10 (22.2)	7 (22.6)	17 (22.4)	0.971
** Neck**	1 (2.2)	0 (0)	1 (1.3)	1.000
** Cheek**	4 (8.9)	6 (19.4)	10 (13.2)	0.326
** Side of nose**	2 (4.4)	1 (3.2)	3 (3.9)	1.000
** Ear**	3 (6.7)	4 (12.9)	7 (9.2)	0.603
** Upper teeth**	2 (4.4)	2 (6.5)	4 (5.3)	1.000
** Lower teeth**	2 (4.4)	2 (6.5)	4 (5.3)	1.000
**Severity (VAS), IQR**	8 (7–10)	8 (7–10)	8 (7–10)	0.900
** Mild (0–3, %)**	0 (0)	1 (3.2)	1 (1.3)	
** Moderate (4–6, %)**	6 (13.3)	4 (12.9)	10 (13.2)	
** Severe (7–10, %)**	39 (86.7)	26 (83.9)	65 (85.6)	
**Quality, n (%)**				
** Stabbing**	22 (48.9)	12 (38.7)	34 (44.7)	0.380
** Electric-shock-like**	13 (28.9)	15 (48.4)	28 (36.8)	0.083
** Shooting**	11 (24.5)	8 (25.8)	19 (25.0)	0.893
** Slashing**	9 (20.0)	5 (16.1)	14 (18.4)	0.669
**Duration, n (%)**				0.302
< **1 s**	0 (0)	2 (6.5)	2 (2.6)	
** 1-600 s**	44 (97.8)	28 (90.3)	72 (94.7)	
> **600 s**	1 (2.2)	1 (3.2)	2 (2.6)	
**Cranial autonomic symptoms, n (%)**				
** Conjunctival injection**	45 (100)	2 (6.5)	47(61.8)	-
** Lacrimation**	45 (100)	29 (93.5)	74(97.4)	-
** Nasal congestion**	10 (22.2)	6 (19.4)	16(21.1)	0.763
** Rhinorrhoea**	17 (37.8)	12 (38.7)	29(38.2)	0.934
** Eyelid oedema**	8 (17.8)	1 (3.2)	9(11.8)	0.117
** Forehead and facial sweating**	5 (11.1)	0 (0)	5(6.6)	-
** Miosis**	0 (0)	0 (0)	0(0)	-
** Ptosis**	3 (6.7)	0 (0)	3(3.9)	-
**Additional symptoms, n (%)**				
** Nausea**	11 (24.4)	4 (12.9)	15 (19.7)	0.214
** Vomiting**	6 (13.3)	2 (6.5)	8 (10.5)	0.562
** Photophobia**	6 (13.3)	3 (9.7)	9 (11.8)	0.902
** Phonophobia**	9 (20.0)	5 (16.1)	14 (18.4)	0.669
** Sense of restlessness and agitation**	8 (17.8)	10 (32.3)	18 (23.7)	0.145

^*^p < 0.05

## Clinical features

### Laterality and location of the attacks

The majority of SUNCT and SUNA patients had strictly unilateral attacks, with a similar ratio of right-side to left-side attacks (20:24 and 15:13, respectively). Only four patients experienced side-alternating unilateral attacks. The involvement of the first branch of the trigeminal nerve (V1), which innervates the periorbital, forehead, and parietal regions and the side of the nose, were most common in our cohort (64.4% in SUNCT patients, 83.9% in SUNA patients), with SUNA patients more likely to have parietal side involvement (*p* = 0.002). Pain in the second branch of the trigeminal nerve (V2), which innervates the temporal region, cheek, and upper teeth, was experienced by 60.0% of SUNCT and 32.3% of SUNA patients, with pain in the temporal region more common in the former patients (*p* = 0.017). Only two patients in each group experienced attacks involving the third branch of the trigeminal nerve (V3), which innervates the lower teeth. In addition, 28.9% of SUNCT patients and 35.5% of SUNA patients experienced attacks outside the trigeminal nerve distribution area (C2–C3), such as the occiput, neck, and ear (Table 1).

### Pain severity and quality

Headache severity was evaluated using the VAS and was rated as severe by both groups (score of 7–10 in 86.7% of SUNCT and 83.9% of SUNA patients). Only a minority of patients reported moderate pain (VAS score of 4–6 in 13.3% of SUNCT and 12.9% of SUNA patients). One SUNA patient had a mild attack (VAS score of 3). There was no discernible difference in pain severity between the two diseases (Table 1). Stabbing pain (44.7%), electric-shock-like pain (36.8%), shooting pain (25.0%), and slashing pain (18.4%) were the most common types of attacks in both SUNCT and SUNA patients (Table 1), with one or more pain features occurring during an episode.

### Duration of attacks

Almost all patients (94.7% of SUNCT and SUNA patients, *n* = 72) reported a headache duration of 1–600 s according to the ICHD-3 criteria. However, attacks lasting > 600 s occurred in one patient with SUNA (up to 900 s) and in one with SUNCT (up to 1,800 s). In these patients, indomethacin-responsive headaches were ruled out due to the effectiveness of therapeutic doses of indomethacin. Two SUNA patients had a self-reported attack duration of < 1 s (Table 1).

### Frequency of attacks

The vast majority of patients in our cohort (75.0%) had a mean attack frequency between 1 and 100 episodes per day. Only 25% of patients (24.4% of SUNCT, *n* = 11; 25.8% of SUNA, *n* = 8) reported an attack frequency of > 100 episodes per day.

### Cranial autonomic symptoms

The cranial autonomic symptoms in our patients included conjunctival injection, lacrimation, nasal blockage, rhinorrhoea, eyelid oedema, and ptosis, which encompassed the common autonomic features of SUNCT and SUNA, except for the absence of miosis in the entire cohort. All SUNCT patients developed conjunctival injection and lacrimation, in keeping with the diagnostic criteria, whereas 93.5% (*n* = 29) of SUNA patients had lacrimation, but only 6.5% (*n* = 2) had conjunctival injection. Only SUNCT patients experienced forehead and facial sweating as well as ptosis. There were no significant differences in the remaining symptoms between patients with SUNA and those with SUNCT (Table 1).

### Attack triggers and aggravating factors

Exclusively spontaneous attacks were reported by 35.6% (*n* = 16) of SUNCT patients and 51.6% (*n* = 16) of SUNA patients. Warning signs of an attack, such as cephalic and facial activities, oral and skin irritation, and odour changes, were reported by 64.4% (*n* = 29) of SUNCT patients and 48.4% (*n* = 15) of SUNA patients. The most common triggers were cold wind (47.8%, *n* = 11), teeth brushing (30.4%, *n* = 7), and face washing (30.4%, *n* = 7) in SUNCT patients and cold wind (54.5%, *n* = 6), face washing (54.5%, *n* = 6), and light touch (54.5%, *n* = 6) in SUNA patients (Table 2). Four patients reported that alcohol consumption was an aggravating factor and one that menstruation was such a factor.

**Table 2:** Attack triggers in patients with SUNCT (*n* = 45) and SUNA (*n* = 31)

	**SUNCT** **n (%)**	**SUNA** **n (%)**	***p* Value (< 0.05)**
**Spontaneous attack**	16 (35.6)	16 (51.6)	0.178
**Triggered attack**			
** Chewing/eating**	6 (13.3)	5 (16.1)	0.434
** Cold wind**	11 (24.4)	6 (19.4)	1.000
** Light touch**	5 (11.1)	6 (19.4)	0.114
** Teeth brushing**	7 (15.6)	5 (16.1)	0.459
** Washing/brushing hair**	1 (2.2)	3 (9.7)	0.089
** Talking**	4 (8.9)	5 (16.1)	0.111
** Face washing**	7 (15.6)	6 (19.4)	0.262
** Swallowing**	2 (4.4)	4 (12.9)	0.070
** Exercise**	1 (2.2)	2 (6.5)	0.239
** Bright lights**	1 (2.2)	1 (3.2)	1.000
** Loud noises**	1 (2.2)	2 (6.5)	0.239
** Strong smells**	1 (2.2)	0 (0)	1.000
** Blowing nose**	1 (2.2)	3 (9.7)	0.089
** Valsalva maneuvers**	1 (2.2)	0 (0)	1.000
** Neck movements**	1 (2.2)	1 (3.2)	1.000

### Refractory period

Among the patients with available data, only 1 of 24 SUNCT patients and 2 of 11 SUNA patients had a refractory period after a cutaneous trigger.

### Previous diagnosis and diagnostic delays

Before their inclusion in the cohort, the patients had been diagnosed with cluster headache (19.7%, *n* = 15), trigeminal neuralgia (11.8%, *n* = 9), tension-type headache (1.3%, *n* = 1), or migraine (1.3%, *n* = 1), but the majority (65.8%, *n* = 50) had been diagnosed with ‘neurovascular headache’ or ‘unknown’. None of the patients had been correctly diagnosed prior to presentation. The median delay to the correct diagnosis was 2.5 (interquartile range: 0–20.5) years for SUNCT patients and 3.0 (interquartile range: 0–20.7) years for SUNA patients (Table 1). The time from symptom onset until the correct diagnosis was 1 year (6.9%) or less (25.0%) in 31.9% (25/76) of all patients. In some patients, the correct diagnosis was not made until 10 years (5.3%) or longer (6.9%) (Table 3).

**Table 3:** Time delay for correct diagnosis of SUNCT and SUNA

	**SUNCT** **n (%)**	**SUNA** **n (%)**	**Total** **n (%)**
**Less than 1 year**	12 (26.7)	7 (22.6)	19 (25.0)
**1 year**	3 (6.7)	3 (9.7)	6 (6.9)
**2 years**	7 (24.1)	4 (12.9)	11 (14.5)
**3 years**	5 (11.1)	0 (0)	5 (6.6)
**4 years**	3 (6.7)	2 (18.2)	5 (6.6)
**5 years**	0 (0)	1 (9.1)	1 (1.3)
**6 years**	0 (0)	1 (9.1)	1 (1.3)
**7 years**	4 (8.9)	1 (9.1)	5 (6.6)
**8 years**	2(4.4)	2 (18.2)	4 (5.3)
**9 years**	2 (4.4)	1 (9.1)	3 (3.9)
**10 years**	4 (8.9)	0 (0)	4 (5.3)
**More than 10 years**	3 (6.7)	3 (9.7)	6 (6.9)

### Treatment effects

A total of 66 patients reported their treatment details and responses; seven received none and three could not recall any treatment. Among them, some patients were effective with NSAIDS (i.e. ibuprofen, diclofenac sodium), compounded painkillers and nasal lidocaine drops. Notably, lamotrigine, topiramate, carbamazepine, duloxetine, gabapentin and pregabalin (usual preventive treatment for SUNCT and SUNA), relieved pain in some patients (Table 4).

**Table 4:** Treatment in patients with SUNCT (*n* = 38) and SUNA (*n* = 28)

	**SUNCT**		**SUNA**	
	Total n (%)	Effectiveness n (%)	Total n (%)	Effectiveness n (%)
**Oxygen**	5 (13.2)	0 (0)	2 (7.1)	1 (3.6)
**Triptan**	2 (5.3)	0 (0)	1 (3.6)	0 (0)
**Indomethacin**	2 (5.3)	0 (0)	2 (7.1)	0 (0)
**Lidocaine**	2 (5.3)	1 (2.6)	0 (0)	0 (0)
**Other NSAIDS**	12 (31.6)	4 (10.5)	5 (17.9)	3 (10.7)
**Combination-analgesic**	14 (36.8)	5 (13.2)	7 (25.0)	3 (10.7)
**Carbamazepine**	4 (10.5)	1 (2.6)	3 (10.7)	1 (3.6)
**Corticosteroids**	3 (7.9)	1 (2.6)	2 (7.1)	0 (0)
**Lamotrigine**	3 (7.9)	2 (5.3)	2 (7.1)	0 (0)
**Topiramate**	3 (7.9)	1 (2.6)	1 (3.6)	1 (3.6)
**Gabapentin**	3 (7.9)	2 (5.3)	3 (10.7)	1 (3.6)
**Duloxetine**	2 (5.3)	1 (2.6)	0 (0)	0 (0)
**Pregabalin**	2 (5.3)	0 (0)	3 (10.7)	2 (7.1)
**Verapamil**	0 (0)	0 (0)	1 (3.6)	0 (0)
**Traditional Chinese medicine**	5 (13.2)	2 (5.3)	5 (17.9)	1 (3.6)
**Anesthetic blockade**	2 (5.3)	0 (0)	1 (3.6)	0 (0)
**Acupuncture**	2 (5.3)	1 (2.6)	2 (7.1)	0 (0)
**Others**	6 (15.8)	0 (0)	2 (7.1)	0 (0)

## Discussion

This is the first multicentre cohort study on SUNCT and SUNA conducted in China. The demographic and clinical characteristics of patients with these uncommon and underreported headaches were determined. Consistent with the literature, the core phenotypes of SUNCT and SUNA were shown to be essentially similar in terms of a female predominance, attack locations concentrated in V1 and V2, and common cranial autonomic symptoms and triggers. However, differences between SUNCT and SUNA were detected, and our results contradicted those of previous studies to some extent.

Our patients with SUNCT and SUNA had an age at onset of around the fourth decade, earlier than in most previous studies [7–10, 14] (Table 5), and similar to the findings of Lambru et al. [11]. Whether the lower age at onset in the Chinese population is due to racial, lifestyle, or cultural factors remains to be determined in studies with larger sample sizes and patients from different regions.

**Table 5:** Clinical characteristics of SUNCT and SUNA from different regions in the world

	China 2022		USA 2021 [14]	UK 2020 [12]	UK 2018 [10]		Portugal 2016 [9]	Australia 2008 [8]		UK 2006 [7]	
	SUNCT	SUNA	SUNCT and SUNA	SUNCT and SUNA	SUNCT	SUNA	SUNCT	SUNCT	SUNA	SUNCT	SUNA
Number of patients	45	31	6	159	65	37	15	17	5	43	9
M: F Ratio	1: 1.14	1: 2.10	1: 1	1: 1.04	1: 0.76	1: 1.06	1: 0.88	1: 1.43	1: 1.5	1: 0.5	1: 2
Age at diagnosis, mean (SD), y	41.62 (12.70)	48.68 (13.80)	NA	58.7 (15.4)	NA	NA	NA	46.24 (11.86)	47.40 (12.72)	NA	NA
Age at onset, mean (SD), y	37.22 (14.54)	42.45 (14.72)	52 (15)	NA	46 (13)	45 (16)	49.7 (12.5)	39.94 (14.44)	46.40 (12.50)	48	44
Common sites of pain (%)	Temporal, periorbital, parietal, forehead	Parietal, temporal. occipital, forehead, cheek	V1 and/ or V2	V1 and/ or V2	NA	NA	Orbital, supra-orbital, temporal	NA	NA	Eye, retro-orbital, forehead, nose	Temple, retro-orbital, side of head
Severity (0-10), mean (SD)	8.13 (1.58)	8.06 (1.83)	NA	9.42 (1.42)	NA	NA	NA	NA	NA	NA	NA
Most cranial autonomic features	Lacrimation, conjunctival injection, rhinorrhoea, nasal congestion	Lacrimation. rhinorrhoea, nasal congestion	Lacrimation, conjunctival injection, rhinorrhoea, facial flushing	NA	Conjunctival injection, lacrimation, nasal blocking or rhinorrhoea, ptosis	Lacrimation, ptosis, nasal blocking or rhinorrhoea, periorbital oedema	Conjunctival injection, lacrimation, ptosis, facial sweating	NA	NA	Conjunctival injection, lacrimation, rhinorrhoea, ptosis	Lacrimation, ptosis, conjunctival injection, rhinorrhoea, nasal blockage
Common triggers	Cold wind, teeth brushing, face washing	Cold wind, face washing, light touch	Teeth brushing, blowing nose	NA	Touch, chewing or eating, wind, brushing the teeth	Chewing, eating, touch	Touching the face or scalp, chewing, eating	Cool breeze from air conditioning ducts	Touch, chew/eat, wind, wash face		Chew/eat, move
Delay to the correct diagnosis, mean (SD), y	2.5 (0–20.5)	3.0 (0-20.7)	NA	NA	NA	NA	NA	NA	NA	6.7	7.1

The female predominance was more pronounced in the SUNCT and SUNA patients of the present study than in most other studies [7–10, 14], consistent with the findings of Lambru et al. [11] (Table 5). An increased proportion of females was also noted among those with cluster headache (CH) [19]. We consider that the female predominance is caused by two factors. First, given the rarity of both diseases, the sex ratios of many previous studies may be inaccurate, given the low numbers of patients; female predominance began to emerge in the present study and that of Lambru et al. [11] (with larger cohorts). Second, similar to CH, social factors such as recent increases in female stress [20] may explain the rise in headache attacks. However, possible evolution of the pathogenesis and regional bias must also be explored. We also found that menstruation was an aggravating factor of attacks, with attacks being more frequent during menstruation in a SUNCT patient. Montes et al. also reported a patient with SUNCT whose attacks were associated with the menstrual cycle, appearing around ovulation. Furthermore, significant relief of the attacks was found to coincide with the end of a pregnancy [21]. These observations suggest that hormones, especially oestrogen, contribute at least partially to the female predominance of SUNCT and SUNA. Support for this hypothesis comes from studies showing effective treatment of refractory SUNCT with clomiphene citrate [22, 23], an oestrogen antagonist that binds to oestrogen receptors, thus also regulating orexin A. Thus, hormone modulation therapy may effectively treat SUNCT, but more data are required.

The reported locations of SUNCT and SUNA attacks vary among studies, but most were in the regions innervated by V1 and V2 (Table 5). SUNCT appeared to involve the temporal region more often, and SUNA the parietal region. However, considering that the temporal and parietal regions share several similar underlying anatomical structures, the differences between them may not be clinically significant. Pain in V3 or outside the trigeminal distribution (C2–C3), such as in the occipital region, was also reported as a radiating location in our cohort, consistent with the findings of Lambru [11] but less commonly described in other studies. This demonstrates the overlap of SUNCT, SUNA and trigeminal neuralgia (TN) in terms of attack location, consistent with previous reports that SUNCT and SUNA attacks mostly involve V1 and V2 and TN attacks V2 and V3 [24, 25]. Some clinicians argue that the boundaries between SUNHA and TN are blurry, and that the two diseases should be considered as a continuum of the same condition with varying degrees of severity [25]. The overlap in the involved locations of the three diseases supports this view.

Among our patients, those with SUNCT had a higher rate and more diverse range of cranial autonomic symptoms than those reported by SUNA patients, and lacrimation was most common in both diseases, consistent with previous studies from Western countries [6–10, 12, 14] (Table 5). However, in the latter, eyelid oedema, ptosis, and miosis were prominent [6, 7, 9, 10, 14], whereas they were less frequent in the SUNCT group and absent in the SUNA group in this study. This discrepancy in the proportion of autonomic symptoms was also a finding of studies of cluster headache, in which the rates of eyelid oedema, ptosis, and miosis were low [26, 27]. The difference might be due to ethnic, social, and cultural differences between Eastern and Western populations.

SUNCT and SUNA can occur spontaneously or can be triggered by cephalic and facial activities, oral and skin irritation, or odour and temperature changes, as determined in our study and previous studies [7–10, 12, 14] (Table 5). The absence of a significant difference in the triggers of SUNCT and SUNA supports the current classification of the two diseases.

Notably, the delay to the correct diagnosis was shorter for SUNCT than SUNA patients, consistent with the findings of Cohen et al. [7]. This difference may reflect the broader and more prominent array of cranial autonomic symptoms of SUNCT, such that individuals who experience these symptoms seek medical help sooner. Because SUNCT is diagnosed based on the presence of both autonomic symptoms of lacrimation and conjunctival injection, according to ICHD-3, while SUNA has greater variability in the range of cranial autonomic symptoms, a diagnosis of SUNA is more likely to be delayed.

### Strengths and limitations

The strengths of our study are that it is the first cohort study of SUNCT and SUNA in China, and was a multicentre work. Thus, the study cohort is representative. We assembled the first clinical cohort of Chinese SUNCT and SUNA patients; our database on Asian patients will aid further investigations in the East. However, this study also had several limitations. First, as it was a cross-sectional study, recall bias was inevitable. Patients with SUNCT and SUNA should undergo regular follow-up visits and maintain a headache diary, as this will aid in the choice of medication and allow an assessment of treatment efficacy. To build on our findings, we have initiated a China SUNCT/SUNA clinical registry study (ChiCTR2200062055) to accumulate data on SUNCT and SUNA via prospective observations of a large number of patients.

## Conclusions

This multicentre clinical study of SUNCT and SUNA is the first of its kind in China, and it established a new cohort for investigating the characteristics of SUNCT and SUNA in the Asian population. Our results as well as those of previous studies revealed statistical differences in some of the clinical features between SUNCT and SUNA, but the shared core phenotypes support the consideration of SUNCT and SUNA as a single clinical entity, in line with the current classification, at least until more data are collected. Definitive conclusions will require a larger number of cases to allow analyses of genetic differences, physiological measurements, and functional imaging.

## Data Availability

The datasets used and/or analysed during the current study are available from the corresponding author on reasonable request.

## Abbreviations

SUNCT: Short-lasting unilateral neuralgiform headache attacks with conjunctival injection and tearing
SUNA: Short-lasting unilateral neuralgiform headache attacks with cranial autonomic symptoms
TAC: Trigeminal autonomic cephalalgia
ICHD: International Classification of Headache Diseases
SUNHA: Short-lasting unilateral neuralgiform headache attacks
VAS: Visual analogue scale
NMOSD: Neuromyelitis optica spectrum disorders
TN: Trigeminal neuralgia
